# A Systematic Review of Transcranial Direct Current Stimulation in Primary Progressive Aphasia: Methodological Considerations

**DOI:** 10.3389/fnagi.2021.710818

**Published:** 2021-10-07

**Authors:** Silke Coemans, Esli Struys, Dorien Vandenborre, Ineke Wilssens, Sebastiaan Engelborghs, Philippe Paquier, Kyrana Tsapkini, Stefanie Keulen

**Affiliations:** ^1^Clinical and Experimental Neurolinguistics, CLIEN, Vrije Universiteit Brussel, Brussels, Belgium; ^2^Center for Neurosciences (C4N), Vrije Universiteit Brussel, Brussels, Belgium; ^3^Department of Speech and Language Pathology, Thomas More University of Applied Sciences, Antwerp, Belgium; ^4^Department of Neurology, Universitair Ziekenhuis Brussel, Brussels, Belgium; ^5^Reference Center for Biological Markers of Dementia, BIODEM, Institute Born-Bunge, Universiteit Antwerpen, Antwerp, Belgium; ^6^Center for Research in Cognition and Neurosciences (CRCN), Université Libre de Bruxelles, Antwerp, Belgium; ^7^Department of Translational Neurosciences (TNW), Universiteit Antwerpen, Antwerp, Belgium; ^8^Department of Neurology, Johns Hopkins School of Medicine, Baltimore, MD, United States; ^9^Department of Cognitive Science, Johns Hopkins University, Baltimore, MD, United States

**Keywords:** primary progressiva aphasia, transcranial direct current stimulation (tDCS), electrode configuration, language rehabilitation, stimulation parameters, speech-and language therapy

## Abstract

A variety of tDCS approaches has been used to investigate the potential of tDCS to improve language outcomes, or slow down the decay of language competences caused by Primary Progressive Aphasia (PPA). The employed stimulation protocols and study designs in PPA are generally speaking similar to those deployed in post-stroke aphasic populations. These two etiologies of aphasia however differ substantially in their pathophysiology, and for both conditions the optimal stimulation paradigm still needs to be established. A systematic review was done and after applying inclusion and exclusion criteria, 15 articles were analyzed focusing on differences and similarities across studies especially focusing on PPA patient characteristics (age, PPA variant, language background), tDCS stimulation protocols (intensity, frequency, combined therapy, electrode configuration) and study design as recent reviews and group outcomes for individual studies suggest tDCS is an effective tool to improve language outcomes, while methodological approach and patient characteristics are mentioned as moderators that may influence treatment effects. We found that studies of tDCS in PPA have clinical and methodological and heterogeneity regarding patient populations, stimulation protocols and study design. While positive group results are usually found irrespective of these differences, the magnitude, duration and generalization of these outcomes differ when comparing stimulation locations, and when results are stratified according to the clinical variant of PPA. We interpret the results of included studies in light of patient characteristics and methodological decisions. Further, we highlight the role neuroimaging can play in study protocols and interpreting results and make recommendations for future work.

## Introduction

### Transcranial Direct Current Stimulation

The effects of transcranial direct current stimulation (tDCS) have initially been extensively investigated for motor-related improvement in healthy (Lang et al., 2004, 2005) subjects and in populations suffering from various acquired and progressive neurological conditions, such as stroke (Fregni et al., 2005; Boggio et al., 2007; Kim et al., 2010), epilepsy (Fregni et al., 2006), Parkinson's disease (Benninger et al., 2010), and multiple sclerosis (Ferrucci et al., 2014). In these studies, different stimulation locations are used according to the nature of the different neurological conditions and the cortical attainment. Research rapidly progressed to studies on cognitive functions such as memory (Galli et al., 2019) and language. tDCS has been shown to improve verbal reaction times (Fertonani et al., 2010), fluency (Cattaneo et al., 2011), word retrieval (Fiori et al., 2011), and grammar learning (de Vries et al., 2010) in healthy subjects, providing a rationale for using tDCS as a tool for rehabilitation in patients suffering from aphasia. As aphasia is commonly seen after stroke, there has been a lot of interest for poststroke language rehabilitation, using tDCS, with promising effects (Hesse et al., 2007; Monti et al., 2008; Baker et al., 2010; Fiori et al., 2011; Fridriksson et al., 2011).

Most study protocols for tDCS in stroke hinge on the recovery model of interhemispheric imbalance (Murase et al., 2004). Many have used anodal tDCS (a-tDCS) on the left perilesional areas (Baker et al., 2010; Fiori et al., 2011; Marangolo et al., 2013a; Campana et al., 2015) to recruit residual neurons in the damaged language areas, departing from the notion that preserved regions are essential to aphasia recovery. Predominantly, the left inferior frontal gyrus (IFG) is stimulated on account of its well-established role in language production and comprehension. Other studies have applied cathodal stimulation to right hemispheric homologous language areas, augmenting left-hemispheric output by disinhibition (Kang et al., 2011; You et al., 2011). Bicephalic montages (Marangolo et al., 2013b, 2014) simultaneously excite left language regions and inhibit right language regions. Positive results have been obtained with a wider extent of electrode montages [for review, refer de Aguiar et al. (2015) and Marangolo (2020)], and the search for the optimal placement of the electrodes is still ongoing (Mahmoudi et al., 2011; Fusco et al., 2013).

A small number of studies have investigated tDCS in progressive aphasias, which will be the subject of this review article.

### Primary Progressive Aphasia (PPA)

Neurodegenerative disorders frequently manifest with syndromes of language deterioration, collectively referred to as PPA. For a diagnosis of a PPA, three criteria must be met as follows: (1) there is a gradual impairment of language, (2) the only plausible cause is a neurodegenerative brain disorder, and (3) aphasia should be the most prominent deficit at the symptom onset and should remain the principal factor impairing daily living activities for at least 1–2 years. The onset usually occurs in the productive years of adulthood, between 40 and 65 years of age (Mesulam et al., 2014), much earlier than is typically seen in other dementias such as Alzheimer's disease (AD), which most commonly occurs after the age of 65 (Mendez, 2019).

There are three commonly accepted clinical variants of PPA according to consensus criteria by Gorno-Tempini et al. (2011): logopenic (phonological) variant of PPA (LvPPA), semantic variant PPA (SvPPA), and nonfluent (agrammatic) variant PPA (NFvPPA). These are summarized in Table 1 (after Gorno-Tempini et al., 2011). Patients can be “unclassifiable” in case they do not display all the necessary features to be assigned to a specific subtype, or can be of a “mixed” type in case criteria belonging to different subtypes are present in one patient. This is true for 15–40% of PPA cases, depending on which criteria are used (Sajjadi et al., 2012; Harris et al., 2013; Wicklund et al., 2014). The mixed phenotype is not yet recognized in the international consensus criteria for PPA classification (Gorno-Tempini et al., 2011). PPA can be linked to several neuropathological changes in the brain. As such, LvPPA is most often associated with AD pathology, while SvPPA and NFvPPA with frontotemporal lobar dementia (FTLD) pathology (respectively FTLD-TDP43 FTLD-tau pathology, specifically). However, there is no one-on-one relationship between neuropathology and the PPA variant (Spinelli et al., 2017). PPA is a severely invalidating condition that leads to limitations in social participation and quality of life, unemployment, and social isolation (Morhardt et al., 2019). Disease-modifying treatments are not yet available for these neurodegenerative brain disorders (Cummings et al., 2018; Panza et al., 2020). Hence, behavioral interventions are the mainstream therapy for patients with PPA. These may consist of two main types: (1) impairment-based speech-and-language therapy and (2) compensatory-based strategies, to maximize functional communication activity and participation levels (Rogalski and Khayum, 2018). Impairment-based studies have mainly addressed lexical retrieval and word fluency, as anomia is a pervasive symptom in all PPA variants (Graham et al., 1999; Croot et al., 2009; Meyer et al., 2015; Tippett et al., 2015; Croot, 2018), and have produced gains in trained words in most studies (Jokel et al., 2014; Volkmer et al., 2020a). These oral and/or written anomia treatments can focus on variant-specific language impairments, i.e., semantically based approaches (e.g., description of use or appearance of an object) for SvPPA (Jokel and Anderson, 2012) or phonological approaches (e.g., provide the first phoneme of the picture name, rhyming) for NFvPPA and LvPPA patients (Jokel et al., 2014). However, mostly, a combination of lexical retrieval therapy approaches has been used, including semantic, phonologic, and orthographic and gestural stimulation, and cueing. Lexical retrieval studies have found significant gains across all subtypes (Henry et al., 2019), although NFvPPA patients seem to benefit more from a phonological than a semantic approach (Jokel et al., 2014). Remarkably, while lexical retrieval difficulties do occur in NFvPPA, it is not a core diagnostic feature in this variant. NFvPPA is defined by agrammatism and apraxia of speech, which can be targeted by grammatical deficit, verb production (Machado et al., 2014; Henry et al., 2018), and script training treatments (Henry et al., 2018). Other speech and language interventions have addressed spelling impairments by focusing on orthographic word forms (Rapp and Glucroft, 2009; Faria et al., 2013; Tsapkini and Hillis, 2013; Marcotte et al., 2014). Refer Jokel et al. (2014) and Volkmer et al. (2020a) for an elaborate review of therapy approaches in PPA. As opposed to poststroke aphasia literature, the impact of nonlinguistic cognitive functions, such as executive functions and planning, has not yet been explored in interventions studies in PPA, although recommended, given the interactions of key non-linguistic functions with the language network (Beales et al., 2018). This is especially true for bilingual aphasia, where switching between languages is thought to be mediated by executive control (Dash and Kar, 2014).

**Table 1:** Classification of primary progressive aphasia (PPA) variants.

	**LvPPA**	**SvPPA**	**NFvPPA**
Clinical features	• Impaired single-word retrieval in spontaneous speech and naming • Impaired repetition of sentences and phrases Additionally, at least 3 of the following features must be present: • Phonologic errors in spontaneous speech and naming • Spared single-word comprehension and object knowledge • Spared motor speech • Absence of outspoken agrammatism	• Impaired confrontation naming • Impaired single-word comprehension Additionally, at least three of the following features must be present: • Impaired object knowledge, particularly for low-frequency items • Surface dyslexia or dysgraphia • Spared repetition • Spared speech production	• Agrammatism in language production • Effortful, halting speech (e.g., apraxia of speech) Additionally, at least two of the following features must be present: • Impaired comprehension of syntactically complex sentences • Spared single-word comprehension • Spared object knowledge
Neuroimaging^*^	• Atrophy is most prominent in the left posterior perisylvian or parietal region on MRI • Hypoperfusion or hypometabolism is most prominent in the left posterior perisylvian or parietal region on SPECT or PET	• Atrophy is most prominent in the anterior temporal lobe on MRI • Hypoperfusion or hypometabolism is most prominent in the anterior temporal region on SPECT or PET	Atrophy is most prominent in the left posterior fronto-insular region on MRI Hypoperfusion or hypometabolism in the left posterior fronto-insular region on SPECT or PET
Most commonly associated pathology	• AD (50-60%)(Mesulam et al., 2008)	• FTLD-TDP (69-83%)(Gorno-Tempini et al., 2011)	• FTLD-tau (52%)(Mesulam et al., 2008)

*PPA, primary progressive aphasia; LvPPA, logopenic/phonological variant PPA; SvPPA, semantic variant PPA; NFvPPA, nonfluent/agrammatic variant PPA; AD, Alzheimer's disease; FTLD-TDP-43, frontotemporal lobar dementia with ubiquitin and transactive response DNA binding protein kDa (TDP-43) pathology; FTLD-tau, frontotemporal lobar dementia with tau-positive pathology*.

* *Disease epicenters. Damage can progress and become more widespread, including white matter (Acosta-Cabronero et al., 2011; Galantucci et al., 2011; Agosta et al., 2012; Mahoney et al., 2013) and functional connectivity (Guo et al., 2013; Agosta et al., 2014; Whitwell et al., 2015; Bonakdarpour et al., 2019) alterations*.

Maintenance and generalizability of gains are still less clear, but a recent systematic review (Cadorio et al., 2017) has concluded that maintenance occurs in all subtypes for at least a short period of time, while generalization (Beeson et al., 2011; Henry et al., 2013a, 2018) seems to vary across PPA subtype, being more likely in LvPPA and NFvPPA than in SvPPA (Graham et al., 1999; Jokel et al., 2002, 2006; Snowden and Neary, 2002; Mayberry et al., 2011; Savage S. A. et al., 2013; Savage S. et al., 2013). This is suggested to be due to connectivity disruptions of the anterior hippocampus in SvPPA (Chapleau et al., 2019); newly learned information can be supported by the non-damaged posterior hippocampus/medial temporal structures but cannot be consolidated effectively to the neocortex (Henry et al., 2019). Maintenance for 6–8 months (Snowden and Neary, 2002; Jokel et al., 2010; Jokel and Anderson, 2012; Henry et al., 2013a,b), 1 year (Henry et al., 2013a, 2018), and 15 months (Meyer et al., 2018, 2019) posttreatment has been documented. However, usually, follow-up is limited.

Research on compensatory-based strategies is much more sparse, although, in reality, often used in actual clinical settings, as speech and language therapists prioritize basic day-to-day communication skills over impairment-based interventions (Kindell et al., 2015; Volkmer et al., 2020b). Strategies to promote successful conversations can mean assessing the strengths and weaknesses of a patient in communication as to develop an adaptive strategy [such as enactment (Kindell et al., 2013)]. It can also focus on communication-partner training and environment support, such as teaching communicative partners facilitative behaviors (e.g., affirmation, less dismissive language) (Taylor-Rubin et al., 2017) and use of assistive devices, e.g., communication books and smartphones (Volkmer et al., 2020a).

### tDCS in PPA

Most studies investigating the effects of tDCS in individuals with PPA have examined whether speech and language therapy effects are augmented when combined with tDCS. In line with tDCS studies in other domains, tDCS studies in PPA have adopted a variety of protocols, leaving some methodological questions about the use of tDCS unanswered. Many different parameters are known to determine the behavioral effects of tDCS, including electrode size and positioning, dosage, polarity, intensity, frequency, and duration (Thair et al., 2017; Vandenborre et al., 2020). Evaluating interactions between these parameters and sorting out their individual effects on behavior outcome, is a complex endeavor. Notwithstanding the discrepancies between the underlying pathologies of acquired and progressive aphasias, studies of tDCS in PPA apply a tDCS montage similar to studies in stroke. In general, two approaches can be identified in poststroke aphasia: stimulation of the lesion site or stimulation of a particular intact node of the language network [cfr. network degeneration hypothesis (Ying, 1996)]. The generalization of these approaches to PPA might not be the most advantageous solution, since patterns of atrophy differ in PPA compared with stroke, and the degenerative nature of the disease leads to progressive changes of neural language representation during the disease. Importantly, an initial modeling study of current flow in the three variants of PPA found that the current flow is distributed in a similar matter as in anatomically typical adults, and local atrophy has no influence on the local electric field (Unal et al., 2020).

Gaining insights into the optimal approach regarding study design and study methodology can help the advancement of the translation of tDCS stimulation to clinical applications. This is especially relevant for PPA, where no pharmacological treatment options for slowing down language decline are at hand. tDCS research in PPA could benefit from more relevant information on the currently less studied variables such as the positions of the electrodes, the effects of tDCS and language therapy in response to the specific deficit of the patients, and effects on functional and structural connectivity.

Our systematic review aims at discussing the methodology of studies on language rehabilitation with tDCS in PPA. We extract patient characteristics (e.g., PPA variant, language background), stimulation parameters (frequency, intensity, and electrode configuration), and study protocol characteristics (language therapy, neuroimaging), and highlight similarities and differences between studies. Next, we discuss these results to provide an assessment of methodological aspects in light of patient characteristics and linguistic outcome measures. Our general goal is to provide an overview and comparison of these characteristics so that methodological aspects in future studies can be improved, and assessments of tDCS effects can become more reliable.

## Methods

### Search Strategies

This review is performed according to the preferred reporting items for systematic reviews and meta-analyses guidelines [PRISMA (Moher et al., 2015)]. The electronic databases Medline (Pubmed) and Web of Science Core Collection were searched for records with terms “(primary progressive aphasia or semantic dementia or logopenic variant PPA or non-fluent variant PPA or semantic variant PPA) and (transcranial direct current stimulation).” Furthermore, bibliographies of retrieved publications were reviewed to identify additional sources (*n* = 0). The date last searched was of July 12, 2021, retrieving 95 publications.

### Study Selection

**Figure 1:**
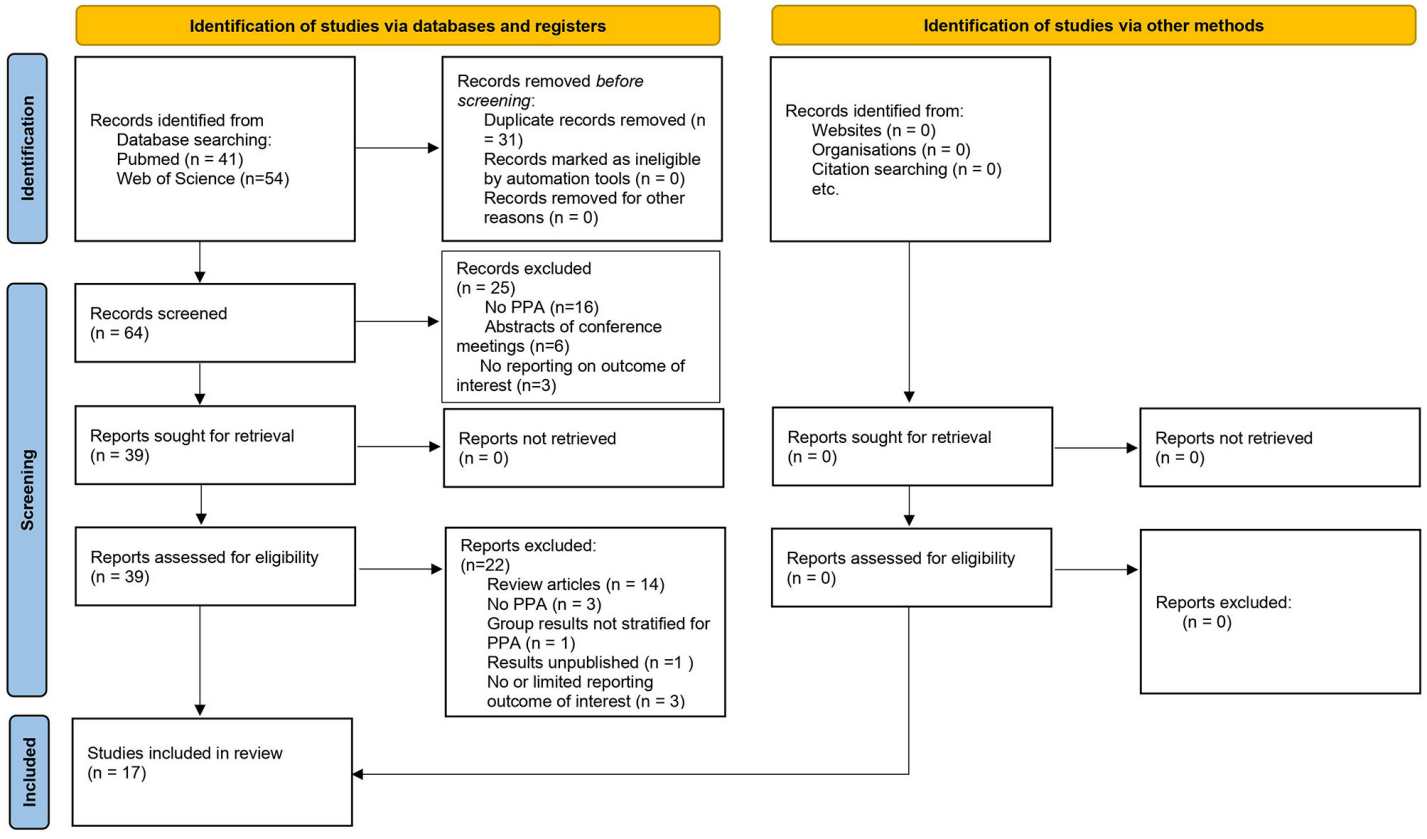
The preferred reporting items for systematic reviews and meta-analyses (PRISMA) diagram of records identified, included, and excluded.

After removal of duplicates (*n* = 31), abstracts were screened (*n* = 64). Exclusion criteria during the screening phase were: study not conducted on patients with PPA (*n* = 16), not using tDCS for speech and language intervention (*n* = 0), abstracts of conference meetings (*n* = 6), or no reporting on language outcome measures (*n* = 3). After the screening phase, original research articles and review articles concerning tDCS to augment language skills in patients with PPA were read full-text and examined in detail (*n* = 39). Studies selected for inclusion in this review (*n* = 17) adhere to the following inclusion criteria: (1) articles written in languages spoken by the authors (English, Dutch, and French), (2) on adults diagnosed with PPA, (3) who received tDCS combined or not combined with speech and language therapy, (3) including all language outcome measures (i.e., oral and/or written naming, grammatical comprehension, and categorization accuracy), (4) and including all types of experimental design, (i.e., case studies, studies without sham-control). Articles excluded after full-text screening were: review articles (*n* = 14), not conducted on patients with PPA (*n* = 3), included other patient populations in group results (*n* = 1), having unpublished results (*n* = 1), no (*n* = 1), or very limited (*n* = 2), reporting on language outcome measures [as shown in Figure 1 (Page et al., 2021)].

### Data Extraction and Analysis

Data were extracted independently by the first author. Baseline information of the studies was extracted, including publication year, protocol design, patient characteristics (age, PPA variant, spoken language(s), duration of disease, pre and posttreatment neuroimaging data). Methodological data were extracted, including the tDCS approach (frequency, duration, intensity, polarity, and location of stimulation), language intervention (type, frequency), and language outcome measures.

## Results

### Patient Characteristics

The ages of patients have high interindividual variability, ranging between 54 and 80 years old. Total group means are similar across the studies, ranging from 66 (SD: 8.3) to 68.7 (SD: 7.0) years old. The average disease duration before the start of the study ranges from 3.6 to 5.3 years, with an average of 4.6 (SD: 0.9) years. Disease severity is usually described as mild to moderate. Studies of tDCS in PPA included a total of 52 SvPPA, 62 LvPPA, 102 NFvPPA, and 8 NFvPPA/apraxia of speech patients. Most studies included a mixed group of PPA. Four studies exclusively included patients with NFvPPA (Wang et al., 2013; Cotelli et al., 2014, 2016). One study exclusively included patients with SvPPA (Teichmann et al., 2016), one study included one patient with LvPPA (de Aguiar et al., 2021), and one study focused on NFvPPA patients with apraxia of speech.

5 studies did not mention the spoken language of the patients (Cotelli et al., 2014, 2016; Roncero et al., 2017; de Aguiar et al., 2020a, 2021) (in these cases, the location where the study was conducted is mentioned in the table). 1 study was performed on a Chinese-speaking patient (Wang et al., 2013), 1 study on native French speakers (Teichmann et al., 2016), and 9 studies included native English speakers (Tsapkini et al., 2014, 2018; Gervits et al., 2016; Hung et al., 2017; McConathey et al., 2017; Fenner et al., 2019; Ficek et al., 2019a; Harris et al., 2019; Themistocleous et al., 2021). Roncero et al. (2019) included fluent French or English speakers (not specifically mentioned whether it was their native language).

### Methodological Approaches

#### Study Design

Studies included either a between-subject sham-controlled condition (Cotelli et al., 2014; Ficek et al., 2019a; Harris et al., 2019; de Aguiar et al., 2020a; Themistocleous et al., 2021) or a within-subject sham-controlled condition (Wang et al., 2013; Tsapkini et al., 2014, 2018; Teichmann et al., 2016; McConathey et al., 2017; Roncero et al., 2017, 2019; Fenner et al., 2019) in their study design, except for Cotelli et al. (2016), Gervits et al. (2016), Hung et al. (2017) and de Aguiar et al. (2021), who did not include a sham-controlled condition. Two studies were case studies (Wang et al., 2013; de Aguiar et al., 2021).

#### Stimulation Parameters

All studies employed conventional tDCS, i.e., two large electrodes placed within presoaked saline sponges, usually with a surface of 5 × 5 cm [with exception of Roncero et al. (2017, 2019)], who used electrodes of 5 × 7 cm), secured to the scalp using a head-strap. The intensity level in all studies was 1–2 mA; stimulation duration lasted at least 20 and a maximum of 30 min. When provided (Cotelli et al., 2014, 2016; Tsapkini et al., 2014, 2018; Hung et al., 2017; Roncero et al., 2017, 2019; Fenner et al., 2019; Ficek et al., 2019a; Harris et al., 2019; de Aguiar et al., 2020a, 2021; Themistocleous et al., 2021) language therapy frequency was identical to tDCS stimulation frequency and lasted for 30-45 min or longer when needed, depending on the capabilities of the individuals. Most studies performed 5 consecutive sessions of tDCS per week, with a total of 10 (Wang et al., 2013; Cotelli et al., 2014, 2016; Gervits et al., 2016; Hung et al., 2017; McConathey et al., 2017; de Aguiar et al., 2020a) sessions per stimulation condition over 2 weeks, or 15 (Tsapkini et al., 2014, 2018; Fenner et al., 2019; Ficek et al., 2019a; Harris et al., 2019; de Aguiar et al., 2020a; Themistocleous et al., 2021) sessions per stimulation condition over 3 weeks. Roncero et al. (2017, 2019) performed 3 sessions of tDCS per week, with a total of 10 sessions per stimulation condition over 3 weeks. Teichmann et al. (2016) was an exception, with 1 session per stimulus condition and sessions separated by 1 week.

#### Electrode Configuration and Language Therapy

Considering the argumentation of electrode placement, 13 of the 17 studies (Cotelli et al., 2014, 2016; Tsapkini et al., 2014, 2018; Hung et al., 2017; Roncero et al., 2017, 2019; Fenner et al., 2019; Ficek et al., 2019a; Harris et al., 2019; de Aguiar et al., 2020a, 2021; Themistocleous et al., 2021) chose their stimulation sites and concordant language therapy based on the predetermined language outcomes under evaluation to enhance task-specific improvements. Teichmann et al. (2016) aspired to compare different electrode montages, specifically for patients with SvPPA, focusing on the main locus of atrophy in these patients. Wang et al. (2013) chose their stimulation sites because of their known role in speech and language. Other studies (Gervits et al., 2016; McConathey et al., 2017) based their electrode montage on inducing a well-distributed current flow throughout the language network.

The spelling intervention of Tsapkini et al. (2014) targeted lexical and sublexical spelling mechanisms in a spell-study-spell procedure (Rapp and Glucroft, 2009) while stimulating the left IFG (F7) with a-tDCS since this area is found to be involved in both lexical (orthographic lexical retrieval) and sublexical (phoneme-to-grapheme conversion) spelling mechanisms. A-tDCS and sham improved spelling outcomes (letter accuracy) in trained items, but effects lasted longer and generalized to untrained items only after a-tDCS. Tsapkini et al. (2018) extended the tDCS spelling protocol to an oral and written naming/spelling paradigm. The paradigm was adapted from a previous behavioral study by Beeson and Egnor (2006)—that had found that integrating semantics, phonology, and orthography may improve lexical access—and incorporated the spell-study-spell procedure by Rapp and Glucroft (2009). Letter accuracy was, again, the outcome measure reported. The left IFG was chosen for its key role in both lexical and sublexical written word production (Purcell et al., 2011). Prompting with semantic features, as in semantic feature analysis, was used as an adjustment to patients who were not able to name the picture, mostly SvPPA patients. The efficacy of tDCS was influenced by the PPA variant: patients with NFvPPA and LvPPA benefited more from a-tDCS and treatment generalized in untrained items but patients with SvPPA benefited only in trained items in the first period of stimulation, but results did not generalize to untrained items. Subsequent studies adapted the same paradigm, stimulating the left IFG during written naming therapy (Fenner et al., 2019; Ficek et al., 2019a; Harris et al., 2019; de Aguiar et al., 2020a), all finding greater and longer-lasting positive effects after a-tDCS compared with sham. Looking at verbs, in particular, written naming outcomes were compared with oral naming outcomes in verbs (Fenner et al., 2019), with bigger improvements in written naming, and effects sustained for up to 8 weeks. In a study on the effects of a-tDCS combined with oral word repetition therapy on sound duration during speech production in patients with NFvPPA/apraxia of speech, the left IFG was stimulated, because of its role in motor planning in speech production. Overall, tDCS combined with speech therapy resulted in significantly shorter sound durations compared to sham stimulation and compared to baseline performance, lasting until 2 months after treatment. Generalization to untrained items was significant immediately after treatment (Themistocleous et al., 2021).

Cotelli et al. (2014, 2016) and Roncero et al. (2017, 2019) conducted an oral picture-naming therapy for anomia. Cotelli et al. (2014, 2016) applied a-tDCS, targeting the left dorsolateral prefrontal cortex (DLPFC, specified as 8 cm frontally and 6 cm laterally away from scalp vertex) in patients with NFvPPA. Naming accuracy improved significantly more after a-tDCS than after sham in trained items, with generalization to untrained items. Roncero et al. (2017) anodally stimulated the left inferior parietal lobe (IPL, P3). The outcome measure was the number of words recalled (spontaneous naming). A-tDCS significantly improved spontaneous oral naming on trained and untrained items (spontaneous naming, number of words recalled), with greater and longer-lasting effects than a sham. Combined with the same naming therapy paradigm, Roncero et al. (2019) compared effects of a-tDCS over P3 and a-tDCS over the left DLPFC (F3) and found improved spontaneous oral naming in trained items for all montages immediately after treatment, with effects 2 weeks after treatment being superior after P3 stimulation. Significant improvement for untrained items was only found after P3 stimulation. Finally, Hung et al. (2017) combined an error-reduced semantic feature analysis training of a target lexicon of ~100 words to each patient with a-tDCS over P3 in patients with LvPPA or SvPPA. Patients showed gains post-tDCS for naming trained items. The authors argue that P3 is important for semantic processing: semantic integration (Price et al., 2016) and semantic working memory (Reilly et al., 2016). One case study performed verbal fluency therapy with orthographic cueing to enhance lexical retrieval, comparing two a-tDCS electrode placements in a within-subject crossover design: stimulation of the left IPL and stimulation of the left IFG. Both stimulations led to improved letter fluency but left IFG simulation gains were greater and generalized to an untrained object-naming task (de Aguiar et al., 2021).

Wang et al. (2013) and Teichmann et al. (2016) did not combine tDCS with language therapy. In an NFvPPA case study, Wang et al. (2013) were not able to include language therapy in their design, as the patient was already in a too-severe stage of the disease. The authors performed a-tDCS stimulation sessions on two different anatomical positions per day: the left posterior perisylvian region in the morning (crossing points T3–P3 and C3–T5) and left Broca's area (crossing point T3–Fz and F7–Cz) in the afternoon. Language outcome measures picture-naming, auditory word identification, oral word reading, and word repetition significantly improved. Teichmann et al. (2016) compared three different montages in SvPPA: cathodal tDCS (c-tDCS) over the right temporal pole (F8–F10) and sham and a-tDCS over the left temporal pole (F7–F9). TDCS was performed for one session for each of these montages. Both tDCS conditions improved comprehension accuracy and processing speed in a semantic-matching task, preceding and immediately following the intervention, with greater effects for right cathodal stimulation.

Gervits et al. (2016) and McConathey et al. (2017) aimed at evaluating a larger set of language functions than previous studies: speech production (words per minute and mean length of utterance), repetition (a sentence-repetition task), grammatical comprehension (correct identification of the target picture of an orally presented sentence) and semantic processing (naming accuracy, categorization accuracy, category-naming fluency). They did not include specific language therapy during tDCS, but patients did have to perform a generic task: freely narrating a story from a picture book of children during stimulation. Both studies opted for a uni-hemispheric montage, providing a-tDCS over the left inferior frontal (F7) and c-tDCS to the left occipito-parietal region (O1). Gervits et al. (2016) found a significant change in performance in 2 out of 4 composite measures: speech production and grammatical comprehension. In contrast to the results from this uncontrolled study, there were no significant improvements found by the within-subject sham-controlled study by McConathey et al. (2017). However, when accounting for baseline performance of the individuals, McConathey et al. (2017) found a stratification of tDCS effects: those with low baseline performance (i.e., more severe aphasia) did show significant improvements, while those with high baseline performance did not.

### Pre/Posttreatment Neuroimaging Studies

6 out of 17 studies did not perform pre (or post) treatment neuroimaging studies (Wang et al., 2013; Cotelli et al., 2014; Tsapkini et al., 2014; Gervits et al., 2016; Hung et al., 2017; McConathey et al., 2017). Tsapkini et al. (2014, 2018), Gervits et al. (2016), Hung et al. (2017), and Themistocleous et al. (2021) reported using the international EEG 10-20 system (Homan et al., 1987) as a tool for anatomical landmarking to guide the placement of the electrodes. Tsapkini et al. (2018) and Themistocleous et al. (2021) also used a fiducial marker to individually coregister the left IFG to pretreament MRI scans.

7 studies (Teichmann et al., 2016; Tsapkini et al., 2018; Ficek et al., 2019a; Roncero et al., 2019, Fenner et al., 2019; Harris et al., 2019 and de Aguiar et al., 2020a) performed pretreatment structural MRI to identify coordinates of the areas to be stimulated. Imaging data, however, were not provided in the publications. One study provided a pretreatment structural MRI that was taken to establish a diagnosis (de Aguiar et al., 2021).

Teichmann et al. (2016) reported on the timing of the MRI scan: less than 1 month prior to treatment. Teichmann et al. (2016) and Roncero et al. (2017) performed FDG-PET scans, respectively, to confirm whether the patients fulfilled the imaging-supported SvPPA diagnosis criteria and to examine hypometabolism in the target region. Cotelli et al. (2016) performed a pretreatment structural MRI scan to evaluate the potential of baseline gray matter density as a predictor of treatment response in patients with NFvPPA. Baseline density of the gray matter of the left fusiform, left temporal, and right inferior temporal gyrus was positively correlated with the improvement in the naming of treated objects. The time between baseline neuropsychological assessment and MRI scan was <2 weeks, while the time between cognitive assessment and beginning of therapy intervention was <3 weeks.

As part of the larger study of Tsapkini et al. (2018), several imaging studies have been performed to evaluate functional connectivity changes caused by tDCS (Ficek et al., 2019a) and to evaluate predictors of success (de Aguiar et al., 2020a; Zhao et al., 2021). Ficek et al. (2019a) performed pre and posttreatment imaging: resting-state functional MRI (fMRI) before, immediately after, and 8 weeks after therapy intervention to evaluate functional connectivity changes modulating tDCS effects. fMRI data were co-registered with the pretreatment structural MRI scans mentioned above to show whether resting-state connectivity changes reflect the structural activity. Improvement in letter accuracy was negatively correlated with functional connectivity between the stimulated site (LIFG, F7) and other posterior areas of the language network compared to sham.

Additionally, an extensive pretreatment neuroimaging study by use of structural MRI was carried out by de Aguiar et al. (2020a), whose aim was to establish gray matter volumetric predictors of tDCS efficacy [next to their study on cognitive predictors (de Aguiar et al., 2020b)]. Greater effects of left IFG stimulation were associated with smaller baseline volumes of brain areas involved in spelling and structurally connected to the left IFG. The amount of atrophy in the IFG itself was not a predictor of stimulation effects. Zhao et al. (2021) next looked at white matter integrity and observed inverse correlations between the integrity of the ventral language pathways and tDCS effects in trained items and between the integrity of dorsal language pathways and tDCS effects in untrained items.

Four studies (Gervits et al., 2016; Teichmann et al., 2016; Roncero et al., 2019; de Aguiar et al., 2020b) reported on their modeled current flow distribution for the chosen electrode montage.

## Discussion

In the current study, we aimed to systematically review the current methods and stimulation parameters of research on the use of tDCS to enhance language abilities in patients with PPA. While some parameters, such as stimulation intensity and frequency of stimulation, did not differ much between studies, other factors, such as the location of stimulation, language therapy, and composition of the study population, did. Most studies support the enhancing effects of tDCS in PPA. A detailed look at the results, however, uncovers a more nuanced picture, with, e.g., differences according to the PPA variant. In the next sections, we interpret the results of included studies in light of possible mediating factors, with a focus on patient characteristics, and methodological decisions. Furthermore, we highlight the role neuroimaging can play in study protocols and how existing evidence of neuroimaging can help with interpreting results.

### Patient Characteristics as Determinants of tDCS Effects

#### Clinical Variant PPA

Group results of Tsapkini et al. (2018) showed tDCS-related improvement in written-naming letter accuracy after stimulation of the left IFG in an oral/written-naming therapy protocol. Crucially, tDCS effectiveness was determined by variant type. Patients with NFvPPA, whose main site of atrophy is the stimulated left IFG, seemed to benefit most from the treatment, i.e., tDCS effects were sustained longer and generalized to untrained items. The left IFG is not the main locus of atrophy in both LvPPA and SvPPA. Patients with LvPPA—whose main loci of atrophy (temporoparietal areas) are connected to the left IFG through the dorsal language stream—did experience generalization of therapy gains after a-tDCS, while patients with SvPPA, with main loci of atrophy connected to the left IFG through the ventral language stream, did not show any generalization to untrained items (although there was a significant tDCS advantage for trained items in the first period of stimulation that was sustained up to 2 months). These results prompt the need to verify tDCS effects on different language hubs for different PPA variants and/or the effects of stimulating specific areas of atrophy in each variant. In addition, the recent meta-analysis by Cotelli et al. (2020) reports greater effect sizes of naming improvement in trained and untrained items for Cotelli et al. (2014, 2016) (stimulation of DLPFC, F3) as compared with Roncero et al. (2017) (stimulation of IPL, P3). Next to possibly being accounted for by the different functional contributions of the stimulated brain areas to the execution of the naming task as discussed in Section tDCS montage and language therapy, the different effect sizes might also be the result of variant-specific effects: Cotelli et al. (2014, 2016) focused on patients with NFvPPA, whose main location of atrophy is the left IFG, an area next to the stimulated DLPFC and probably stimulated given the large size of conventional electrodes. Roncero et al. (2017), on their part, included a mixed group of patients in their study. The parietotemporal montage effects appeared to differ across variants, with 4 out of 4 patients with NFvPPA and only 1 out of 4 patients with SvPPA benefitting from a-tDCS over sham. Thus, their smaller effect size on group results might have been driven by PPA subtypes. Patients with SvPPA suffer more atrophy in the anterior temporal lobe (ATL), a node possibly not adequately activated by tDCS over neither the left IFG, the DLPFC, or the inferior parietotemporal cortex. Teichmann et al. (2016) focused on the main locus of atrophy in patients with SvPPA in a within-subject sham-controlled design, finding significant improvement in a semantic-matching task. However, as Tsapkini et al. (2018) also pointed out in this regard, positive results for SvPPA by Teichmann et al. (2016) were found on a comprehension accuracy task with a 50% chance of success, while the assessments of the studies by Tsapkini et al. (2018) and Roncero et al. (2019) were more complex production tasks. Hung et al. (2017) reported patients with SvPPA to have greater improvements in oral naming accuracy than the other variants after excitatory left temporoparietal stimulation. Considering the lack of a sham control condition, it is, however, not possible to know whether treatment gains are attributable to either tDCS or their semantic anomia treatment, or synergistic effects.

It is important to note that, in prior studies without tDCS intervention, patients with SvPPA were often also found to be less prone to improvement and generalization of results, likely due to their loss of semantic knowledge. It has been suggested repeatedly that generalization in patients with SvPPa should not be expected, unless when semantic impairment is still relatively absent (Bier and Macoir, 2010; Jokel et al., 2014; Krajenbrink et al., 2020). Finally, the study population of Gervits et al. (2016), existed out of 4 patients with LvPPA and 2 patients with NFvPPA, vs. 6 patients with NFvPPA and 1 patient with LvPPA in McConathey et al. (2017). Perhaps, their contrasting results reflect their different patient population compositions. Positive group results of these studies might mask variant-related effects. Individual data could indicate whether all patients benefitted from the adopted methodology and, thus, highlight how stimulation (or other) parameters have differential effects on the clinical PPA variants. There is a need to consider how patient characteristics, such as PPA variant, can affect the choice of a stimulation site and/or language therapy and the eventual therapy outcomes. Unfortunately, due to generally small sample sizes in studies on PPA, results are rarely given stratified per variant [a commonly found stumbling block in aphasia literature, refer Basso (2005).

#### Language Background

The reviewed studies do not always elaborate on the language backgrounds of patients. When mentioned, the patients in each study all share their native language, with the exception of Roncero et al. (2019), who included patients fluent in French or English. Unfortunately, it is not clarified whether the subjects were native speakers and whether they were fluent in either language or, perhaps, were bilingual speakers of French and English. However, the relevance of native language in language processing in healthy individuals and neurological patients is suggested by previous studies: structural or morphological distinctions in language, such as in Hebrew (Bick et al., 2011) or Chinese (Khachatryan et al., 2016) as compared with English, modulate the activation of language areas in the brain in healthy subjects. Furthermore, a recent study has found differences in speech production between monolingual English and Italian patients with NfvPPA, suggesting dementia seems to express itself differently according to the language of the patient (Canu et al., 2020). In writing, differences exist between alphabetic and ideographic writing scripts (Vandenborre et al., 2015), and organization of neural networks subserving graphomotor processing and the underlying cognitive system may vary to different degrees across script systems and scripts (Bolger et al., 2005; Vandenborre et al., 2015).

Establishing the language background of the patients can help clarify the response of the individuals to tDCS and is necessary to take into consideration for evaluation and therapy in neurodegenerative disorders. Regarding bilingual patients, it is known that bilingualism is mediated by structural and functional changes in the brain, leading to neural differences between bi-and monolinguals (Abutalebi et al., 2008; Green and Abutalebi, 2013; Li et al., 2014; Calabria et al., 2018). When deciding on tDCS methodology, differences between bi-and monolinguals need to be considered: e.g., when the stimulation location is chosen based on the language function to be trained or assessed, how can one be sure of its structural/functional position in the bilingual brain? Furthermore, taking differences in patterns of language impairments into account and knowledge of which language may or may not be better preserved can be determining for assessments, therapy, and stimulation.

#### Post-onset Timeframe of Stimulation

Disease severity (i.e., worse brain atrophy levels and more cognitive decline) and baseline performance (i.e., performance on language tests before study-related tDCS and/or speech and language therapy) appear to be predictors of tDCS efficacy. Altogether, current literature suggests that higher atrophy and, thus, more loss of function and poorer baseline language scores make for greater potential for functional improvement. For instance (Wang et al., 2013), where the patient was in a later stage of the disease, with anomia too severe to receive naming therapy: despite disease severity and lack of language therapy intervention, the patient improved on several language outcomes. Furthermore, in the imaging study by de Aguiar et al. (2020a), the greatest improvement was associated with smaller volumes of several brain regions involved in language (e.g., left angular gyrus, middle frontal gyrus, supramarginal gyrus, posterior cingulate cortex). This can have implications on the choice of the timeframe of stimulation, as these results suggest higher efficacy of tDCS in patients who have worse baseline performance and more atrophy, thus, in later stages of the disease. Considering the effects of tDCS on neuroplasticity and its potential to improve the strength of the connections in neurons, still relatively spared in earlier stages, however, early intervention might still be effective and desirable. Neuroimaging evidence could help elucidate the influence of baseline severity as a predictor of response by taking into account the levels of atrophy of the stimulated area and its connected regions, as discussed in Section Neuroimaging to determine underlying mechanisms of treatment and predict treatment effects. Considering treatment interventions with or without tDCS, in general, we would like to point out neuroimaging studies in PPA, indicating that early language treatment intervention is indispensable; for instance, Meyer et al. (2017) suggested that higher levels of atrophy reduce the potential for speech and language therapy benefits and Zhao et al. (2021) positively correlated white matter integrity with treatment outcomes in the sham condition.

### Methodological Characteristics

#### tDCS Montage and Language Therapy

Most studies applied a-tDCS over left hemisphere regions, to increase cortical excitability. The stimulation site was usually chosen in accordance with the language skill targeted during language therapy, irrespective of patient- or variant-specific characteristics. Although the precise mechanisms may vary, it is hypothesized that functional specificity of tDCS may be achieved by activity-selectivity, meaning tDCS may preferentially modulate brain networks that are engaged during stimulation. Thus, tDCS may produce more substantial or targeted effects when combined with functional tasks or treatments (Bikson et al., 2013). When provided, language therapy is usually aligned with the cognitive-linguistic treatment approach, as is often seen in aphasia research. This type of treatment is aimed at phonological and/or semantic processing to restore the linguistic levels affected: semantics, phonology, or syntax (Nouwens et al., 2017). For this, the therapies in the studies reviewed consisted of naming therapy (oral and/or written naming), with protocols enabling co-activation of semantic and phonological (Roncero et al., 2017, 2019), or semantic, phonological and orthographic presentations (Cotelli et al., 2014, 2016; Tsapkini et al., 2014, 2018; Fenner et al., 2019; Ficek et al., 2019a; Harris et al., 2019; de Aguiar et al., 2020a). The study by Themistocleous et al. (2021) was the only study that employed speech therapy focused on speech production specifically, finding positive effects of stimulating the left IFG, critical to motor planning in speech production, on normalizing sound durations in NFvPPA patients with apraxia of speech. This use of cognitive-linguistic treatment approaches is in line with the literature on speech and language therapy in PPA without tDCS (Henry and Grasso, 2018). The second approach in aphasia treatment is communicative treatment: treatment focusing on compensatory strategies and use of residual language skills by, for instance, script training and training of priority vocabulary (de Jong-Hagelstein et al., 2011). In clinical practice, speech and language therapists report using communicative treatment more often than a cognitive-linguistic (naming) treatment approach, related to the frequent disengagement of patients from naming therapies (Croot, 2018; Volkmer et al., 2018). Hung et al. (2017) chose a maintenance approach rather than a restorative approach, aiming at the preservation of known words instead of restoration of lost vocabulary. A priority vocabulary of 100 words, together with personalized cues (photographs of the items), was crafted in conjunction with the patients. Using autobiographic cues to take advantage of the relatively spared episodic memory to aid word retrieval has previously proved to be successful for SvPPA (Henry et al., 2013b). While studies mostly targeted naming abilities, different locations known to be involved in oral/written naming were chosen for stimulation: the left IFG, DLPFC, and the left IPL. Despite the varying positions of the anodal or cathodal electrode, all studies, with the exception of one (McConathey et al., 2017), reported tDCS-related improved language outcomes [refer to meta-analyses (Byeon, 2020; Cotelli et al., 2020)] for effect sizes). The electrode montage of McConathey et al. (2017) study was identical to Gervits et al. (2016), who did find positive treatment effects in measures of speech production and grammatical comprehension. This might indicate the relevance of other methodological parameters that did differ, such as a presence of a within-subject sham-controlled condition, while there was a lack of a control condition in Gervits et al. (2016) (Cotelli et al., 2016; other studies without sham control: Hung et al., 2017).

Interestingly, the notion that different electrode montages do lead to similar outcome effects prompted (Roncero et al., 2019) to directly compare a-tDCS over the left IPL (as in Roncero et al., 2017) with a-tDCS over the left DLPFC (as in Cotelli et al., 2014, 2016). Both montages, combined with repeated naming therapy sessions, yielded beneficial results compared with sham regarding the number of words recalled. Furthermore, the type of stimulation received did have an impact on the performance: for trained items, improvements immediately after treatment were similar after DLPFC and IPL stimulation, but 2 weeks after treatment, improvements were greater after IPL stimulation compared with DLPFC and sham stimulation. For untrained items, IPL stimulation was the only montage where significant improvement was found. These results might reflect the stimulated different functions of areas: the role of the DPLFC in working memory may have supported training effects, leading to more short-term improvement in trained items. Stimulation of the IPL on its part may have exerted its longer-lasting and more generalized effects through the role of supramarginal gyrus in phonological processing and the involvement of the angular gyrus in semantic integration functions (Price et al., 2016; Hartwigsen, 2018). These functions support naming task performance and are known to fall short in PPA variants [i.e., dorsal (phonological) system and ventral (semantic) system (Henry et al., 2016)]. However, only quantitative, not qualitative information on naming accuracy, was provided. In a verbal fluency task, the overall number of words generated is determined by both clustering (the production of words in a subcategory) and switching (switching to a new subcategory). Montage setups might affect these outcomes, with DLPFC stimulation supporting executive control and, thus, switching abilities, and temporal stimulation supporting lexical contribution by clustering. Qualitative measures of this task could provide valuable insights into the effects of tDCS on linguistic and executive control strategies in patients with PPA, and how these are related to areas of stimulation (Troyer et al., 1998). The case study by de Aguiar et al. (2021) also compared 2 different electrode montages during verbal fluency therapy: stimulation of the left IFG and stimulation of the left IPL. Both setups led to an increase in the amounts of words retrieved during a letter fluency task, but gains were greater and generalized to an untrained object-naming task only after stimulation of the left IFG. This study is an exception when it comes to therapy, opting for verbal fluency speech therapy rather than traditional naming therapy. This more challenging task might prove to be useful for patients with milder anomia, for which oral/written picture-naming tasks do not pose great difficulty. Together, the results of Roncero et al. (2019) and de Aguiar et al. (2021) suggest that stimulating different nodes in one particular network can lead to different results, and that, despite the non-focality of tDCS, the location of stimulation might be a variable critical to success.

While Wang et al. (2013) were not able to include speech and language therapy because of the disease severity of their patient, the authors did find improvement of several language skills after a-tDCS. Furthermore, 3 studies chose not to combine tDCS with speech and language therapy, with 2 of them (Gervits et al., 2016 and Teichmann et al., 2016) reporting tDCS-related improvements in language outcomes. Instead of language therapy, the authors did instruct their patients to perform a small task during stimulation: Gervits et al. (2016) and McConathey et al. (2017) instructed patients to narrate a situational scene, a task to engage the language network. The patients of Teichmann et al. (2016) performed a visuomotor task during tDCS to remain vigilant. These studies, however, have some important limitations: the study of Wang et al. (2013) was a case study, thus inherently lacking external validity. Gervits et al. (2016) did not include a control sham condition. Importantly, the effects observed by the pilot study by Gervits et al. (2016) could not be replicated in the subsequent randomized sham-controlled design by McConathey et al. (2017). Other than emphasizing the need for a sham condition in future research, the absence of tDCS-related improvements could be caused by the lack of a more specific task during stimulation. Evidence from the literature currently suggests synergic effects of pairing tDCS with behavioral treatment, a plausible consequence of the neuromodulatory nature of tDCS effects on the membrane-resting potential (Monti et al., 2013; Price et al., 2015; Holland et al., 2016). In PPA, a recent study has found enhanced performance after tDCS in the therapy task only, clarifying that the correct choice of language intervention performed during stimulation might be a determinative factor in achieving the intended outcomes (Bikson et al., 2013; Ficek et al., 2019a).

#### Neuroimaging to Determine Underlying Mechanisms of Treatment and Predict Treatment Effects

TDCS studies in PPA address behavioral effects of the therapy intervention, but, to date, little is known about the underlying neural mechanisms of tDCS. Neuroimaging [e.g., DTI or (f)MRI] studies can help to identify and understand the affected neural circuits. Through brain networks, these circuits can extend beyond the site of stimulation. When effectuated pre and posttreatment, neuroimaging can be utilized to investigate brain changes underlying behavioral tDCS effects and eventually establish where and how stimulation can exert its effects on brain function. Furthermore, neuroimaging can be used as a predictor of success and/or can aid the selection of montage placement: structurally by identifying the target region, or functionally by measuring neural activation to localize the cognitive function of interest. Functional neuroimaging might have an added value to disentangle the regions a particular patient uses for a particular language task. For example, in a post-stroke tDCS study, pretreatment fMRI was conducted to pinpoint the brain region that was most active during a naming task, which was then stimulated during therapy (Baker et al., 2010). The reviewed studies of tDCS in PPA have mainly chosen their stimulation site in relation to the particular language task given, thereby targeting active brain regions to increase stimulation efficacy. This rationale is supported by evidence suggesting tDCS efficacy seems to depend on previous neural activity and the task at hand (Bikson et al., 2013). Therefore, functional targeting may, indeed, prove to be more fruitful than perilesional stimulation, where stimulation effects might be lost or diminished because of structural and/or functional deterioration. Pretreatment functional imaging could aid in choosing these functional target sites, as in Baker et al. (2010).

In terms of the underlying neuronal mechanisms of tDCS in PPA, Ficek et al. (2019a) identified a possible mechanism of altered functional connectivity: the authors performed functional MRI pre and posttreatment (after sham and after real tDCS) imaging and found that letter accuracy was correlated with a decrease in functional connectivity (compared with sham) between the stimulated left IFG and posterior areas of the language network. One possible explanation provided is that abnormalities in baseline connectivity in PPA between frontal and temporal areas may be regulated by tDCS. A recent study has examined the changes in the functional reorganization of hubs in PPA (Tao et al., 2020). They showed that all variants lost hubs in the left superior frontal and parietal regions, while new hubs were recruited in different areas. The functional reorganization was not fully accounted for by local structural changes, as was the case in a prior study by Mandelli et al. (2018), who found functional changes before noticeable volume loss. Next to further exploring the effects of delivering stimulation directly to atrophied areas, it might be fruitful to elucidate the role of other (functional) language hubs. Studies on healthy aging populations (Meinzer et al., 2013) and in mild cognitive impairment (Meinzer et al., 2015), have found correlations between positive behavioral effects of tDCS with decreased functional connectivity as well. Pretreatment structural MRI was used in several studies to localize target area coordinates, but this imaging data were not provided in the publications. Information about atrophy in the targeted, or connected, areas could elucidate the differential responses of patients and/or the PPA variants to treatment, as well as identify patients who will respond favorably to stimulation in one well-determined area. Furthermore, consideration of interindividual differences in structural or functional connections of the brain is required for an individualized application of tDCS.

Cotelli et al. (2016), de Aguiar et al. (2020a), and Zhao et al. (2021) performed pretreatment structural MRI. To evaluate gray matter density as a predictor of tDCS efficacy in patients with NFvPPA, Cotelli et al. (2016) performed a pretreatment structural MRI scan. The authors did not report whether imaging data were used to support the choice of electrode positioning, or on how stimulation location was determined. Results indicated that improvement of the naming of treated objects was positively correlated with gray matter density in the left fusiform, right inferior temporal, and left temporal gyrus. Improvements in the naming of actions were correlated with gray matter density in the left middle temporal gyrus. These areas remain largely spared in the early phases of NFvPPA, suggesting that partially spared language-related areas are essential for therapy-induced language improvement and intervention in early disease stages might be best. de Aguiar et al. (2020a) established an extensive pretreatment neuroimaging study by use of structural MRI. The authors concluded that greater effects of left IFG stimulations were associated with smaller baseline volumes of brain areas involved in spelling (the primary outcome measure) and structurally connected to the left IFG, such as the left angular gyrus, left supramarginal gyrus, and left middle frontal gyrus. The amount of atrophy in the IFG itself was not a predictor of stimulation effects. In line with Ficek et al. (2019a), these results suggest that tDCS may induce functional changes and that mainly regions with greater atrophy (and, perhaps, more functional distortions) have a greater potential for functional improvement. The importance of, specifically, the integrity of regions connected to the stimulated left IFG clarifies the need to unravel relevant contributions of different nodes of the language network. Given the assumed role of tDCS in plasticity, DTI can be an important tool to visualize structural remodeling of tissue and thus investigate the translation of therapy to neuroplasticity in terms of brain volume increase or decrease. DTI, for example, could be used to analyze the effects of tDCS on the structural degeneration of white matter pathways (Galantucci et al., 2011). Zhao et al. (2021) looked at structural connectivity before and after 3 weeks of tDCS but did not find any changes in white matter. As 3 weeks might be too short a period of time for volume changes to occur, it would be interesting to see whether structural neuroimaging at a later point in time would show white matter changes. Zhao et al. (2021) did find white matter integrity to be a predictor of language therapy without tDCS as well as of language therapy with tDCS, in line with the study by de Aguiar et al. (2020a) on gray matter density as a predictor of tDCS effects. Depending on white matter integrity was greater for language therapy without tDCS, with less damage to the white matter being associated with better language therapy outcomes. Language therapy with tDCS on its part was inversely correlated with white matter integrity: more disintegration of white matter in dementia-related areas leads to greater tDCS effects, suggesting tDCS is more effective in severer stages of the disease. This is in line with McConathey et al. (2017), who found baseline performance to be inversely correlated with therapy outcomes. Their results also coincide with the functional connectivity study by Ficek et al. (2019a), and the authors conclude by suggesting that white-matter integrity may be a mediator of functional connectivity as a mechanism of tDCS effects. These studies clearly show how structural and functional imaging evidence is essential to assess the ability of tDCS to induce effects on these nodes and projection pathways in the language network and of the underlying mechanisms (i.e., functional connectivity).

Gervits et al. (2016), Teichmann et al. (2016), Tsapkini et al. (2018), Fenner et al. (2019), Ficek et al. (2019a), Harris et al. (2019), Roncero et al. (2019), and de Aguiar et al. (2020a) reported on their modeled current flow distribution for the chosen electrode montage to predict peak areas of activation and adequate distribution of current through the language network. Computational current flow modeling can aid in approximating the optimal stimulation site and reduce the variability of the spread and intensity of the current, caused by differences in brain morphology (e.g., cerebrospinal thickness, cortical folding), electrical properties of the tissue, and cerebrospinal fluid (Cancelli et al., 2015; Evans et al., 2020). Importantly, a recent modeling study on the impact of brain atrophy on tDCS current flow in all 3 PPA variants has found that current delivery to the brain is not substantially altered in patients with PPA compared with healthy individuals. Hence, the authors suggest that an individualized electrode montage for delivering the appropriate dose is not necessary for PPA (Unal et al., 2020). While current flow studies can be helpful, they still have limitations (Rudroff et al., 2020) and cannot fully account for the interindividual variability. Combined use of tDCS, neuroimaging, and computational studies may be required for the optimization of tDCS in PPA.

#### Washout Period

In studies with a crossover study design, an interval between real and sham tDCS stimulation phases is necessary to avoid carryover effects, which can lead to false-positive or false-negative results. The washout period for tDCS is not well understood yet. Wang et al. (2013) did introduce a washout period of 1 week between each week of 5 consecutive sessions of a-tDCS and sham interventions (with 20 sessions in total). Significant outcomes were only found after the first tDCS phase. Thus, the patient may have reached a ceiling effect during the first period of intervention, with aftereffects of real tDCS outlasting the remaining experimental protocol.

In other studies, a washout period of 8 weeks was introduced after 15 (Tsapkini et al., 2014, 2018; Fenner et al., 2019) and 10 (Roncero et al., 2019) consecutive tDCS sessions. Tsapkini et al. (2014, 2018) reported that results for trained items significantly improved after the first real tDCS phase compared with sham, but these results were not apparent after the second (sham) phase. It is possible that tDCS effects were carried over into the subsequent sham phase for some patients, leading to a greater sham performance in the second phase. If a ceiling effect is reached during the first period of the intervention, the second period of intervention offers less room for improvement. Fenner et al. (2019) also reported diminishing of tDCS effects compared with sham in the second phase. To investigate tDCS in a crossover design, a longer washout period may be necessary, as for, instance, 3 months such as in Gervits et al. (2016), McConathey et al. (2017), and Themistocleous et al. (2021). However, considering the progressive nature of the disease, a complete washout might not be desirable. To measure carryover effects, one might consider providing additional assessments in between phases of tDCS stimulation.

## Conclusion and Future Directions

Studies of tDCS in PPA have clinical and methodological and heterogeneity regarding patient populations (PPA variant, language background, severity, etc.), and stimulation protocols (electrode configuration, combined language therapy), and study design. Although positive (group) results are usually found irrespective of these differences, these parameters might impact the effectiveness of tDCS in PPA. Questions remain as to the optimal parameters for the most effective use of tDCS, such as stimulation of which area of the brain is more effective for improving language skills in patients with PPA. While all stimulation locations, thus far, have produced positive outcomes, the magnitude, duration, and generalization of these outcomes differ when comparing stimulation locations, and they differ when stratified for PPA variant. When choosing an area of stimulation, it might be crucial to pair this with the language therapy of choice. Most studies have combined tDCS with language therapy, usually focusing on naming tasks. This combination of tDCS with a relevant task has been proposed to maximize the impact of tDCS, with the neuromodulatory nature of tDCS facilitating the effects of therapy through neuronal activation, and vice versa (Ficek et al., 2019a). Optimizing tDCS parameters can also mean searching for other possible candidates for the site of stimulation. For instance, the cerebellum is relatively spared in patients with PPA and is involved in several language skills (Marien et al., 2014), and its stimulation has led to positive results in poststroke aphasia (Sebastian et al., 2020).

Other questions related to patient characteristics and their role in the efficacy of tDCS, such as PPA variant and language background (e.g., spoken language, bilingualism) of the patients. To determine which individuals will benefit more from tDCS intervention and to explain heterogeneity in treatment effects, the development of biomarkers can be helpful. Several types of biomarkers are potential targets to help determine predictors of tDCS success, such as genetic studies, cerebrospinal fluid analytes, characterization of cognitive and language profiles of the patients, and neuroimaging (Grossman, 2014). Neuroimaging can, for instance, reveal anatomical correlates of success, i.e., brain volumes of the stimulated area or task-relevant networks (de Aguiar et al., 2020b). On the other hand, neuroimaging work can help to evaluate structural or functional connectivity changes occurring after stimulation of a certain target area.

## Data Availability Statement

Publicly available datasets were analyzed in this study. This data can be found here: https://www.sciencedirect.com/science/article/pii/S0304394013005661?via%3Dihub, ScienceDirect https://content.iospress.com/articles/journal-of-alzheimers-disease/jad131427, IOS Press https://www.ncbi.nlm.nih.gov/pmc/articles/PMC4470615/, PMC, PMC4470615 https://link.springer.com/article/10.1007/s10548-016-0494-2, Springer https://www.ncbi.nlm.nih.gov/pmc/articles/PMC5204261/, PMC, PMC5204261 https://www.ncbi.nlm.nih.gov/pmc/articles/PMC5492829/, PMC, PMC5492829 https://onlinelibrary.wiley.com/doi/full/10.1002/ana.24766, Wiley https://www.frontiersin.org/articles/10.3389/fnins.2019.01231/full, Frontiers in Neuroscience https://www.ncbi.nlm.nih.gov/pmc/articles/PMC5651421/, PMC, PMC5651421 https://www.frontiersin.org/articles/10.3389/fnhum.2017.00253/full, Frontiers in Neuroscience https://www.sciencedirect.com/science/article/pii/S2213158218301682?via%3Dihub, Elsevier https://www.ncbi.nlm.nih.gov/pmc/articles/PMC6153381/, PMC, PMC6153381 https://www.ncbi.nlm.nih.gov/pmc/articles/PMC6582664/, PMC, PMC6582664 Sciencedirect https://www.sciencedirect.com/science/article/pii/S0197458019300892?via%3Dihub; https://www.sciencedirect.com/science/article/pii/S0093934X19303232?via%3Dihub, Sciencedirect https://www.tandfonline.com/doi/full/10.1080/02687038.2021.1881432, Taylor & Francis Group https://www.mdpi.com/2076-3425/11/3/335, MDPI.

## Author Contributions

SK, ES, and SC contributed to the conception and design of the review. SC collected the data, performed the analysis, and wrote the paper. KT, DV, and IW wrote sections of the manuscript. All authors contributed to several manuscript revisions and read and approved the submitted version.

## Funding

SC was supported by the Research Foundation—Flanders (FWO), Grant No. FWOAL938-Junior Research Project. KT was supported by grants from the Science of Learning Institute at Johns Hopkins University and by the NIH/NIDCD through award R01 DC014475 and NIH/NIA through award R01 AG068881.

## Conflict of Interest

The authors declare that the research was conducted in the absence of any commercial or financial relationships that could be construed as a potential conflict of interest.

## Publisher's Note

All claims expressed in this article are solely those of the authors and do not necessarily represent those of their affiliated organizations, or those of the publisher, the editors and the reviewers. Any product that may be evaluated in this article, or claim that may be made by its manufacturer, is not guaranteed or endorsed by the publisher.
