# Supplementary material for: A Systematic Review of Transcranial Direct Current Stimulation in Primary Progressive Aphasia: Methodological Considerations
Source: Front Aging Neurosci. 2021 Oct 7;13:710818. doi: 10.3389/fnagi.2021.710818 (PMC8530184; doi:10.3389/fnagi.2021.710818)
Supplement: Supplementary file 1 [file Table_1.docx]

**Supplementary Table 1: Review of methodological characteristics and language outcomes of studies on tDCS in PPA.**

| **Study** | **Participants** | **Age, mean (SD)** | **Language spoken**  **(Location)** | **Time post-onset (years)** | **Protocol Design** | **Interphase interval** | **Anode** | **Cathode** | **tDCS approach: intensity, duration, sessions** | **Language therapy** | **Evaluation, time post-treatment** | **Primary outcome measures** | **Results on primary outcome measures** | **Pre-post treatment neuroimaging** |
| --- | --- | --- | --- | --- | --- | --- | --- | --- | --- | --- | --- | --- | --- | --- |
| Wang et al. (2013) | 1 NFvPPA | 67 | Chinese | 5 | Sham-controlled case-study | 1 week | Morning: Left posterior perisylvian region (T3-P3 x C3-T)5 Afternoon: left Broca’s area (T3-Fz x F7-Cz) | Right shoulder | 1.2 mA, 20 min, 5 sessions sham (phase A1; A2) and tDCS (phase B1; B2), each phase over 5 days in an A1-B1-A2-B2 design | NA | T1: Immediately post- treatment | Auditory word-picture identification, picture naming, oral word reading, word repetition | Significant improvement of auditory word-picture identification, picture naming, oral word reading, word repetition after a-tDCS, not after sham. Decline 8 weeks post-treatment. | None |
| Cotelli et al. (2014) | 16 NFvPPA | Total group: 66.9 (8.2)  atDCS group: 63.4 (6.8)  Sham tDCS group: 70.4 (6.8) | NA  (Brescia, Italy) | NA | Randomized between-subject controlled design | NA | Left dorsolateral prefrontal cortex (8 cm frontally and 6 cm laterally away from Cz) | Right arm | 2 mA, 25 min, 10 sessions sham or tDCS over two weeks | 25 min speech therapy during a-tDCS: production of target noun by (1) repetition of target word, (2) articulatory suppression, (3) oral picture naming, (4) reading target word | T1: Immediately post-treatment  T2:12 weeks post-treatment | Percentage of correct responses in an experimental naming assessment for trained and untrained stimuli  Number of correct responses in untrained naming subtest of Aachener Aphasie Test. | Percentage of correct responses in experimental naming assessment improved significantly for trained and untrained items after a-tDCS and sham tDCS at T1 and T2 but this effect was significantly greater after a-tDCS for trained items.  Number of correct responses on the naming subtest of the Aachener Aphasie Test increased selectively after a-tDCS, with effects remaining significant at T2.  Other:  Analysis of daily living language abilities improved after a-tDCS, but not after sham. | None |
| Tsapkini et al. (2014) | 2 NFvPPA, 4 LvPPA | NA | Native English speakers | NA | Randomized within-subject controlled design | 8 weeks | Left anterior temporal region: left IFG (F7) | Right cheek | 1-2 mA, 20 min, 15 sessions tDCS over 3 weeks, 15 sessions sham, each over 3 weeks | 30 min spelling intervention during a-tDCS: treatment of the phoneme to-grapheme conversion mechanism, whereby patients had to write a letter or letter combination corresponding to (a) given phoneme(s) | T1: Immediately post-treatment  T2: 2 weeks post-treatment  T3: 8 weeks post-treatment | Accuracy initial phoneme/grapheme of each word and the word prompt as a whole; number of words spelled correctly | Significant improvements of written naming accuracy of trained items after tDCS and sham stimulation at T1, T2 and T3, with effects significantly greater for a-tDCS than sham.  Significant improvement s of written naming accuracy of untrained items lasting until T3 after a-tDCS. | None |
| Cotelli et al. (2016) | 18 NFvPPA | 66.5 (9.5) | NA  (Brescia, Italy) | NA | Case series pre-post design  No sham condition | NA | Left dorsolateral prefrontal cortex (8 cm frontally and 6 cm laterally away from Cz) | Right shoulder | 2mA, 25 min, 10 sessions a-tDCS over two weeks | 25 min speech therapy during a-tDCS: production of target noun by (1) repetition of target word, (2) articulatory suppression, (3) oral picture naming, (4) reading target word | T1: Immediately post-treatment T2: 12 weeks post-treatment | Percentage of correct responses in an experimental naming assessment for trained and untrained stimuli  Number of correct responses in untrained naming subtest of Aachener Aphasie Test. | Percentage of correct responses in experimental naming assessment improved significantly for trained and untrained items after a-tDCS at T1 and T2, with greater effects for trained items.  Number of correct responses on the untrained naming subtest of the Aachener Aphasie Test improved after a-tDCS at T1. | Pre-treatment MRI three weeks prior to therapy, to evaluate the potential of grey matter density as a predictor of treatment response.  Improvement of naming of trained items was positively correlated with baseline GM volume in the left fusiform, left middle temporal and right inferior temporal gyri. |
| Gervits et al. (2016) | 2 NFvPPA, 4 LvPPA | 66.2 (5.7) | Native English speakers | 4.2 (1.8) | Case series pre-post design  No sham condition | NA | Left anterior temporal region: left IFG (F7) | Left occipitoparietal region (O1) | 1.5 mA, 20 min, 10 sessions simultaneous a-tDCS and c-tDCS, over two weeks | NA | T1: Immediately post-treatment  T2: 6 weeks post-treatment  T3: 12 weeks post-treatment | Speech production (composite score of speech rate and mean length of utterance measures from the Cookie Theft task), sentence repetition (total number of completely correct repeated sentences) grammatical comprehension (number of correct identifications target picture of orally presented sentence), semantic processing (composite score of naming accuracy, categorization accuracy, category naming fluency) | Significant improvements on composite measures of speech production at T1 and grammatical comprehension at T1, T2 and T3, non-significant improvements on sentence repetition and composite measure of semantic processing.  Significant improvements in global performance (average of all four of the composites) at T1, T2, T3. | None |
| Teichmann et al. (2016) | 12 SvPPA | SvPPA group: 66.8 (2.1)  Healthy control group: 64.1 (7.4) | Native French speakers | 5.3 (0.8) | Randomized within-subject controlled design | 1 week | Phase 1: Left temporal (FT7 to FT9)  Phase 2: left supra-orbital  Phase 3: sham left temporal | Phase 1: Right supra-orbital  Phase 2: Right temporal (FT8 to FT10)  Phase 3: sham right supra-orbital | 1.59 mA, 20 min, 3 sessions over three weeks (one session per montage condition) | NA |  | Percentage of correct responses and reaction times on a semantic matching task, including an orthographic and a visual item representation modality of living and non-living items. | Left a-tDCS and right c-tDCS significantly improved percentage of correct responses in the orthographic modality of the semantic matching task.  Right c-tDCS additionally significantly improved reaction times on questions regarding living items. | Pre-treatment MRI and PET-scan <1 month prior to therapy, respectively to localize target area coordinates and to confirm SvPPA diagnosis. |
| Roncero et al. (2017) | 6 NFvPPA, 2 LvPPA, 2 SvPPA | Total group: 67.4 (5.9)  NFvPPA: 69.0 (5.8)  SvPPA: 63 (7.5)  AD: 67.3 (3.1) | NA  (Montréal, Canada) | NA | Randomized within-subject controlled design | 8 weeks | Left inferior parietotemporal region: left IPL(P3) | Right fronto-orbital region | 2mA, 30 min, 10 sessions a-tDCS and 10 sessions sham, each over 18 days | Speech therapy during a-tDCS: Picture naming /repeated naming of missed (incorrectly named) at that day’s session. Sessions lasted at least the length of tDCS stimulation (30 min), and until all missed items were trained, but no longer than 2 h. | T1: Immediately post-treatment T2: 2 weeks post-treatment | Number of correct responses during oral naming of trained and untrained items (whole words) | Improvements of number of correct oral naming responses of trained items after a-tDCS and sham, with significant grater effects after a-tDCS compared to sham at T1 and T2.  Improvements of number of correct oral naming responses of untrained items only after a-tDCS.  Other: Scores on Mini-Mental State Examination, Montreal Cognitive Assessment and verbal fluency remained unchanged. | Pre-treatment FDG-PET scan at time of enrolment to document level of left temporo-parietal hypometabolism.  Pre-treatment structural MRI to localize target area coordinates. |
| Hung et al. (2017) | 1 LvPPA, 3 SvPPA, 1 early onset AD | Total group: 66.6 (8.56)  SvPPA: 67.3 (8.7)  LvPPA: 71  AD: 60 | Native English speakers | Total group:  3.6 (1)  SvPPA: 3.7 (1.2) | Case series pre-post design  No sham condition | NA | Left temporoparietal region: left IPL (P3) | Center of forehead | 1.5 mA, 20 min, 10 a-tDCS sessions over 2 weeks | 30 min semantic feature analysis on 21 target words per day during a-tDCS: Repeated spontaneous naming, sentence production, semantic feature generation | T1: Immediately post-treatment  T2: 24 weeks post-treatment | Number of correct responses during oral naming of trained and untrained nouns (whole words) | A-tDCS improved naming accuracy for trained items at T1 compared to baseline, but not at T2.  There were no improvements for untrained items. | None |
| McConathey et al. (2017) | 6 NFvPPA, 1 LvPPA | 68.7 (7.0) | Native English speakers | 4.3 (1.9) | Randomized within-subject controlled design | 12 weeks | Left anterior temporal region: left IFG (F7) | Left occipitoparietal region (O1) | 1.5 mA, 20 min, 10 sessions sham OR tDCS over two weeks | NA | T1: Immediately post-treatment  T2: 6 weeks post-treatment  T3: 12 weeks post-treatment | Speech production (composite score of speech rate and mean length of utterance measures from the Cookie Theft task), sentence repetition (total number of completely correct repeated sentences) grammatical comprehension (number of correct identifications target picture of orally presented sentence), semantic processing (composite score of naming accuracy, categorization accuracy, category naming fluency) | No significant improvements on composite measures of speech production, sentence repetition, grammatical comprehension, or semantic processing. | None |
| Ficek et al. (2018) | 8 NFvPPA, 8 SvPP, 8 LvPPA | Total group 67.2 (6.5)  tDCS (n=12) 65.2 (7.0)  Sham (n=12) 69.1 (5.6) | Native English speakers | Total group: 4.9 (3.0)  tDCS: 5.5 (3.5)  Sham: 4.3 (2.4) | Randomized between-subject controlled design | NA | Left anterior temporal region: left IFG(F7) | Right cheek | 2.0 mA, 20 min, 15 sessions sham OR a-tDCS over three weeks | 40-45 min oral and written naming/spelling therapy during tDCS | T1: Immediately post-treatment | Written letter accuracy of trained words | Significant improvements written letter accuracy of trained items after sham and a-tDCS, with significant greater effects after a-tDCS relative to sham. | Pre-and posttreatment fMRI before, immediately after and 8 weeks after therapy to evaluate functional connectivity changes. Co-registered with structural MRI. |
| Tsapkini et al. (2018) | 14 NFvPPA, 12 LvPPA, 10 SvPPA | NFvPPA: 70 (5.8)  SvPPA: 68.6 (5.2)  LvPPA: 65.3 (8.4) | Native English speakers | NA | Randomized within-subject controlled design | 8 weeks | Left anterior temporal region: left IFG(F7) | Right cheek | 2.0 mA, 20 min, 15 sessions a-tDCS and 15 sessions sham, each over three weeks | 40-45 min oral and written naming/spelling therapy during a-tDCS | T1: Immediately post-treatment T2: 2 weeks post-treatment  T3: 8 weeks post-treatment | Percentage of correct letter-to sound responses (letter accuracy) in an oral and written naming (spelling) task of trained and untrained words. | NFvPPA and LvPPA:  Improvement letter accuracy trained and untrained items after a-tDCS and sham, with greater effects for a-tDCS than sham, and lasting until T2 after a-tDCS.  SvPPA:  No greater improvements in letter accuracy after a-tDCS compared to sham. | Pre-treatment structural MRI to localize target area coordinates. |
| Roncero et al. (2019) | 4NFvPPA, 4 LvPPA, 4 SvPPA | Total group: 65.4 (6)  NFvPPA: 70.3 (4.9)  LvPPA 64.3 (5.9)  SvPPA: 61.8 (6.2) | Fluent French or English speakers | NA | Randomized within-subject controlled design | 8 weeks | Comparison three different electrode montages: (1) a-tDCS left inferior parietotemporal region: left IPL (P3), (2) sham tDCS left inferior parietotemporal region (P3), (3) a-tDCS left DLPFC (F3) | (1) right supra-orbital, (2) right supra-orbital, (3) right deltoid muscle. | 2mA, 30 min, 10 sessions a-tDCS and 10 sessions sham, each over 18 days | Speech therapy during a-tDCS: Picture naming /repeated naming of missed (incorrectly named) at that day’s session.  Sessions lasted at least the length of tDCS stimulation (30 min), and until all missed items were trained, but no longer than 2 h. | T1: Immediately post-treatment  T2: 2 weeks post-treatment  T3: 8 weeks post-treatment | Number of words recalled during oral naming assessment of trained and untrained nouns (whole words) | DLPFC and left inferior parietotemporal stimulation improved number of words recalled of trained items significantly larger than sham at T1, T2 and T3 with superior improvements after parietotemporal at T2.  Number of words recalled for untrained items deteriorated after sham, but remained stable after a-tDCS over DLPFC. Parietal-temporal montage yielded significant benefit for untrained items at T2.  Effects were most evident in NFvPPA and least evident in SvPPA.  Other:  Scores on Mini-Mental State Examination, Montreal Cognitive Assessment, verbal fluency and digit span remained unchanged. | Pre-treatment structural MRI to localize target area coordinates. |
| Fenner et al. (2019) | 6 NFvPPA,  5 LvPPA | tDCS: 67.6 (7.7)  Sham: 70.5 (5.0) | Native English speakers | tDCS: 5 (3.5)  Sham: 6.3 (2.8) | Randomized within-subject controlled design | 8 weeks | Left anterior temporal region: left IFG (F7) | Right cheek | 2.0 mA, 20 min a-tDCS, 10-14 sessions tDCS and 10-14 sessions sham, each over three weeks | 40-60 min verb therapy during a-tDCS: spell-study-spell procedure, oral and written naming paradigm. | T1: Immediately post-treatment T2: 2 weeks post-treatment  T3: 8 weeks post-treatment | Written letter accuracy of trained and untrained verbs, evaluated at single word, not sentence | Significantly larger improvements of written letter accuracy of trained verbs after a-tDCS than after sham at T1 For untrained verbs, there were significantly larger improvements of written letter accuracy after a-tDCS compared to sham at T1 and T3. | Pre-treatment structural MRI to localize target area coordinates. |
| Harris et al. (2019) | 10 NFvPPA, 6 SvPPA, 6LvPPA | Total group: 66.9 (7.5)  tDCS: 64.1 (8.4)  sham: 69.6 (5.7) | English as first language | Total group: 5.0 (3.0)  tDCS: 5.6 (3.4)  Sham: 4.5 (2.5) | Randomized between-subject controlled design | NA | Left anterior temporal region: left IFG (F7) | Right cheek | 2.0 mA, 20 min, 15 a-tDCS sessions or 15 sham sessions over three weeks | 45-50 oral and written naming therapy during atDCS | T1: Immediately post-treatment  T2: 8 weeks post-treatment | Percentage of correct letter-to sound responses (letter accuracy) in an oral and written naming (spelling) task. | Letter accuracy improved significantly after a-tDCS and sham, at T1 and T2, with greater effects after a-tDCS than sham.  Other:  In tissue targeted by a-tDCS, GABA levels reduced, lasting for 8 weeks. | Pre-treatment structural MRI to localize target area coordinates. |
| de Aguiar, Zhao, Faria, et al. (2020) | 9 NFvPPA,  7 SvPPA,  14 LvPPA | Total group: 66.4 (6.7)  TDCS: 64.3 (7.4)  sham: 68.8 (5.1) | NA  (Baltimore, United States) | 4.7 (3.0) | Randomized between-subject controlled design | NA | Left anterior temporal region: left IFG (F7) | Right cheek | 2.0 mA, 20 min, 15 a-tDCS sessions or 15 sham sessions over three weeks | 45-50 min oral and written naming therapy during a-tDCS | T1: Immediately post-treatment T2: 2 weeks post-treatment  T3: 8 weeks post-treatment | Percentage of correct letter-to sound responses (letter accuracy) in an oral and written naming (spelling) task. | Letter accuracy of trained items improved significantly  more after a-tDCS than sham at T3, but not at T1 or T2.  Letter accuracy for untrained items improved significantly more after a-tDCS than sham at T1 and T3. | Pre-treatment structural MRI to extract baseline brain volumes and to localize target area coordinates |
| De Aguiar et al. (2021) | 1 LvPPA | 72 | NA | NA | Single-case alternating treatment design | 14 weeks | Phase 1: Left anterior temporal region: left IFG (F7)  Phase 2: left inferior parietotemporal region: left IPL (P3) | Right cheek | 2.0 mA, 20 min, 10 a-tDCS sessions over two weeks for each phase | 60 min Letter Fluency Therapy during a-tDCS: retrieval of as many words as possible starting with each of the trained letters, in periods of 1-3 min | Left IFG: T1: Immediately post-treatmentT2: 2 weeks post-treatment  T3: 14 weeks post-treatment  Left IPL: T1: Immediately post-treatmentT2: 2 weeks post-treatment  T3: 10 weeks post-treatment | Number of words retrieved during letter fluency for trained and untrained letters  Changes in naming accuracy in an untrained object naming task | Number of words retrieved per trained letter increased significantly from baseline after left IFG stimulation at T1 and T2, and left IPL stimulation at T1, with greater effects after left IFG stimulation.  Generalization to untrained letters was significant for both stimulation locations at T1, T2 and T3.  Changes in naming accuracy in untrained object naming task only occurred after left IFG stimulation. | Pre-treatment structural MRI to establish diagnosis |
| Themistocleous, Webster, Tsapkini (2021) | 8 NFvPPA/AOS | Total group:  66 (8.3)  Sham group: 67.67 (5.27)  tDCS group: 65 (7.64) | Native English speakers | Total group: 3.3 (1.4)  Sham group: 2.7 (1.3)  tDCS group: 3.7 (1.48) | Randomized between-subject controlled design | NA | Left anterior temporal region: left IFG (F7) | Right cheek | 2.0 mA, 20 min, 15 a-tDCS sessions OR 15 sham sessions over three weeks | 45 min speech therapy during sham or tDCS: oral word repetition of increasingly morphologically complex words | T1: Immediately post-treatment T2: 8 weeks post-treatment | Vowel and consonant sound duration | Sound duration was significantly shorter after tDCS compared to baseline and compared to sham for trained items at both evaluations and for untrained items immediately post-treatment. This was true for both vowels and consonants. | Pre-treatment structural MRI to localize target area coordinates. |
